# Supplementary material for: A yeast phenomic model for the influence of Warburg metabolism on genetic buffering of doxorubicin
Source: Cancer Metab. 2019 Oct 23;7:9. doi: 10.1186/s40170-019-0201-3 (PMC6806529; doi:10.1186/s40170-019-0201-3)
Supplement: Supplementary file 9 — Additional file 9. GO term-specific heatmaps for REMc/GTF-enriched clusters. GO term-specific heatmaps for significant GO process terms were generated as described in methods and Figs. 3 and 4. Any related child terms are presented in subsequent pages of the parent file name. GO terms with more than 100 children, with 2 or fewer genes annotated to the term, or a file size over 300KB are not shown. All heatmaps are generated with the same layout (see Figs. 3 and 4). [file 40170_2019_201_MOESM9_ESM.bz2 › Additional_File9_GOTermHeatmaps/Additional_File9_GOTermHeatmap/RNA_splicing,_via_transesterification_reactions.pdf]

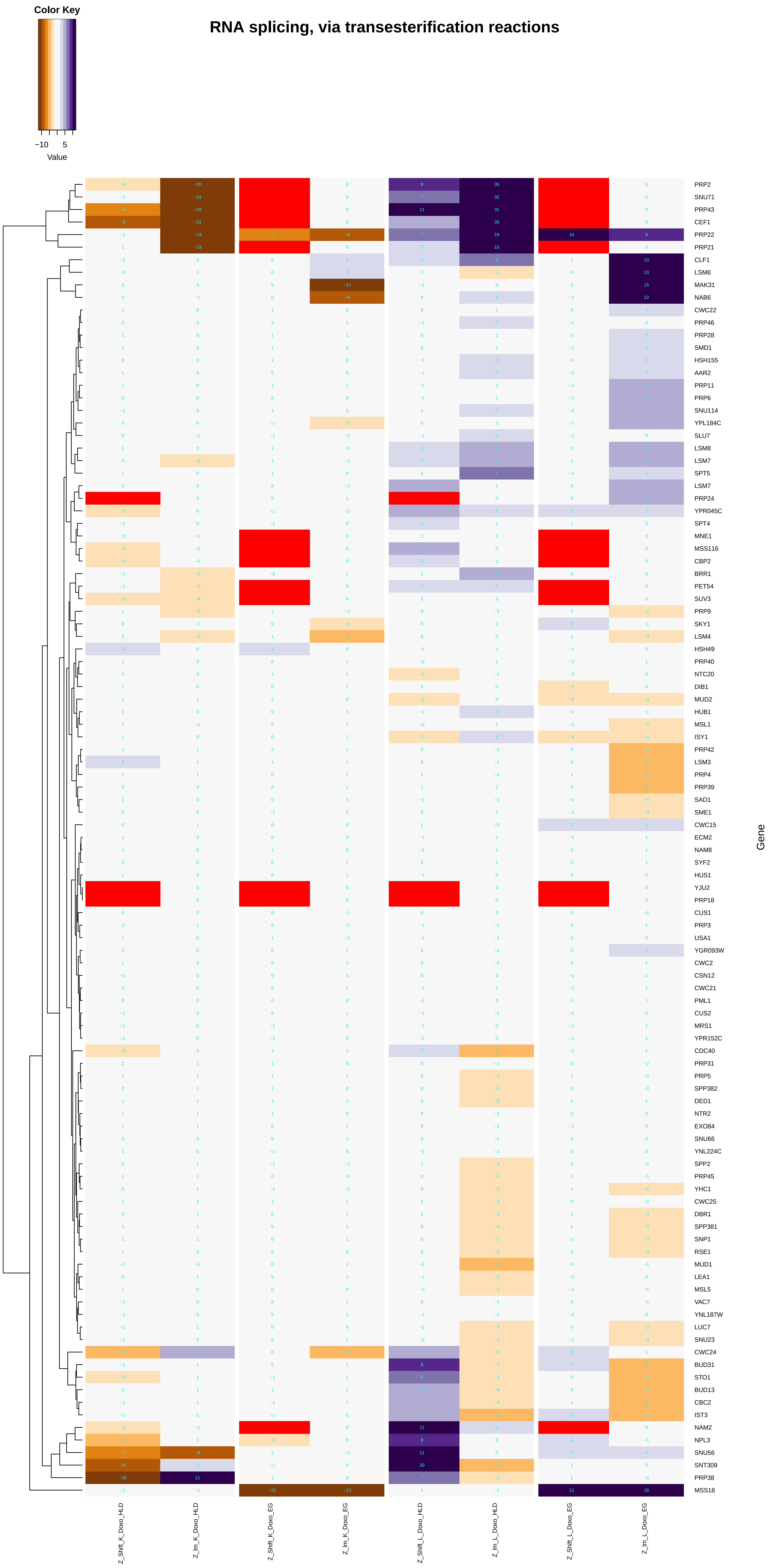
RNA splicing, via transesterification reactions
Color Key
−10
5
Value
PRP2
SNU71
PRP43
CEF1
PRP22
PRP21
CLF1
LSM6
MAK31
NAB6
CWC22
PRP46
PRP28
SMD1
HSH155
AAR2
PRP11
PRP6
SNU114
YPL184C
SLU7
LSM8
LSM7
SPT5
LSM7
PRP24
YPR045C
SPT4
MNE1
MSS116
CBP2
BRR1
PET54
SUV3
PRP9
SKY1
LSM4
HSH49
PRP40
NTC20
DIB1
MUD2
HUB1
MSL1
ISY1
PRP42
LSM3
PRP4
PRP39
SAD1
SME1
CWC15
ECM2
NAM8
SYF2
HUS1
YJU2
PRP18
CUS1
PRP3
USA1
YGR093W
CWC2
CSN12
CWC21
PML1
CUS2
MRS1
YPR152C
CDC40
PRP31
PRP5
SPP382
DED1
NTR2
EXO84
SNU66
YNL224C
SPP2
PRP45
YHC1
CWC25
DBR1
SPP381
SNP1
RSE1
MUD1
LEA1
MSL5
VAC7
YNL187W
LUC7
SNU23
CWC24
BUD31
STO1
BUD13
CBC2
IST3
NAM2
NPL3
SNU56
SNT309
PRP38
MSS18
Gene
Z_Shift_K_Doxo_HLD
Z_Im_K_Doxo_HLD
Z_Shift_K_Doxo_EG
Z_Im_K_Doxo_EG
Z_Shift_L_Doxo_HLD
Z_Im_L_Doxo_HLD
Z_Shift_L_Doxo_EG
Z_Im_L_Doxo_EG

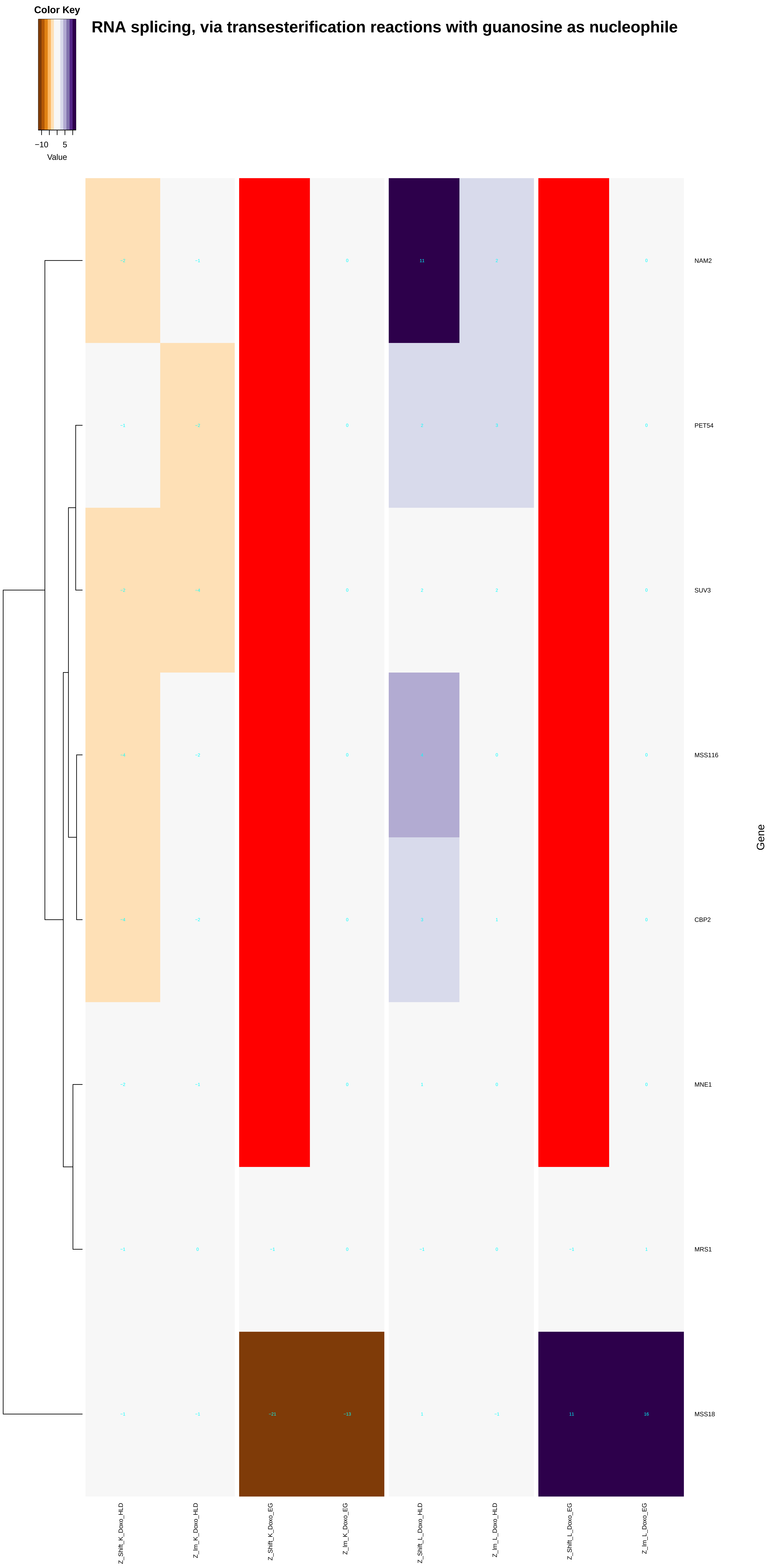
Color Key
RNA splicing, via transesterification reactions with guanosine as nucleophile
−10
5
Value
NAM2
PET54
SUV3
MSS116
CBP2
MNE1
MRS1
MSS18
Gene
Z_Shift_K_Doxo_HLD
Z_lm_K_Doxo_HLD
Z_Shift_K_Doxo_EG
Z_lm_K_Doxo_EG
Z_Shift_L_Doxo_HLD
Z_lm_L_Doxo_HLD
Z_Shift_L_Doxo_EG
Z_lm_L_Doxo_EG

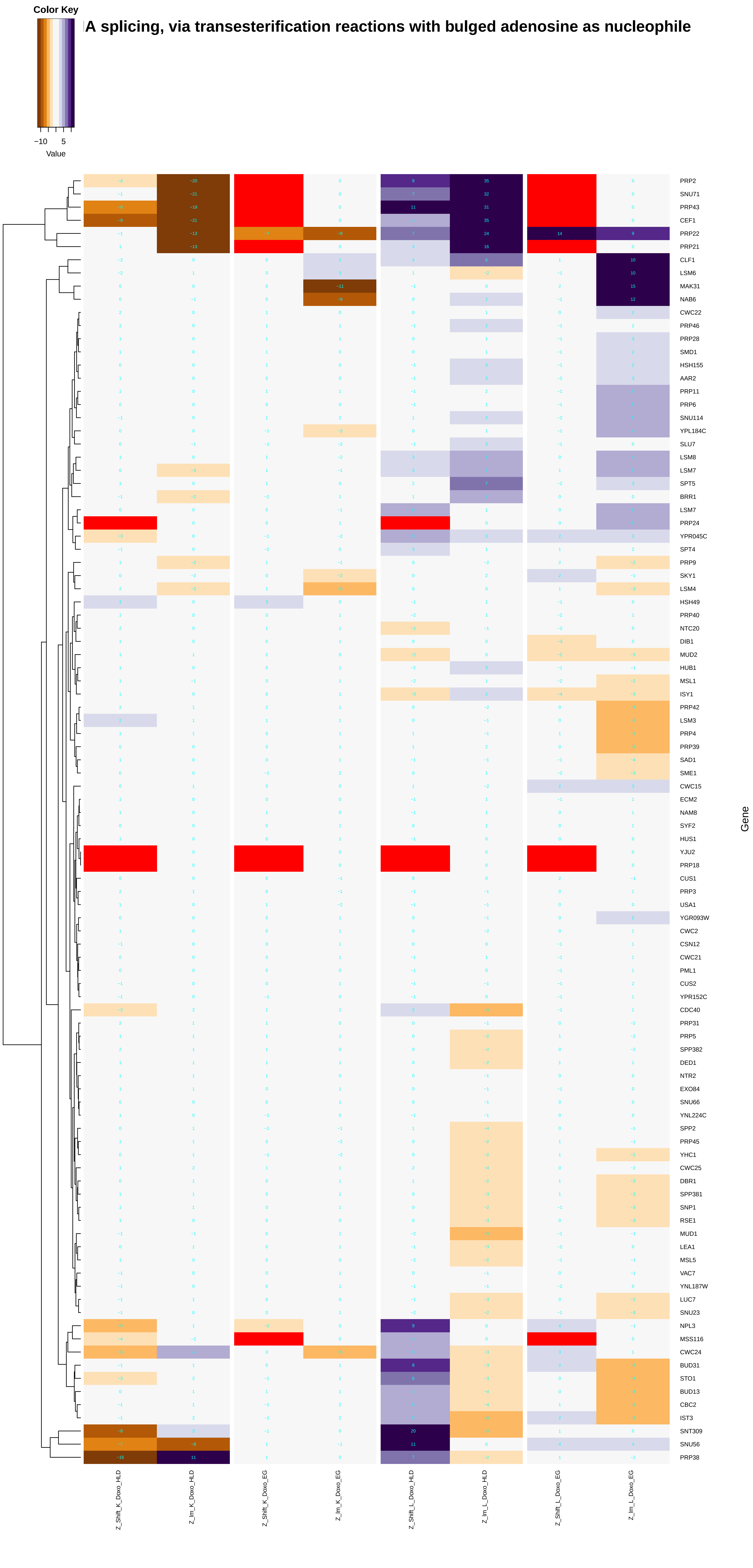
Color Key
A splicing, via transesterification reactions with bulged adenosine as nucleophile
−10
5
Value
Gene
PRP2
SNU71
PRP43
CEF1
PRP22
PRP21
CLF1
LSM6
MAK31
NAB6
CWC22
PRP46
PRP28
SMD1
HSH155
AAR2
PRP11
PRP6
SNU114
YPL184C
SLU7
LSM8
LSM7
SPT5
BRR1
LSM7
PRP24
YPR045C
SPT4
PRP9
SKY1
LSM4
HSH49
PRP40
NTC20
DIB1
MUD2
HUB1
MSL1
ISY1
PRP42
LSM3
PRP4
PRP39
SAD1
SME1
CWC15
ECM2
NAM8
SYF2
HUS1
YJU2
PRP18
CUS1
PRP3
USA1
YGR093W
CWC2
CSN12
CWC21
PML1
CUS2
YPR152C
CDC40
PRP31
PRP5
SPP382
DED1
NTR2
EXO84
SNU66
YNL224C
SPP2
PRP45
YHC1
CWC25
DBR1
SPP381
SNP1
RSE1
MUD1
LEA1
MSL5
VAC7
YNL187W
LUC7
SNU23
NPL3
MSS116
CWC24
BUD31
STO1
BUD13
CBC2
IST3
SNT309
SNU56
PRP38
Z_Shift_K_Doxo_HLD
Z_lm_K_Doxo_HLD
Z_Shift_K_Doxo_EG
Z_lm_K_Doxo_EG
Z_Shift_L_Doxo_HLD
Z_lm_L_Doxo_HLD
Z_Shift_L_Doxo_EG
Z_lm_L_Doxo_EG

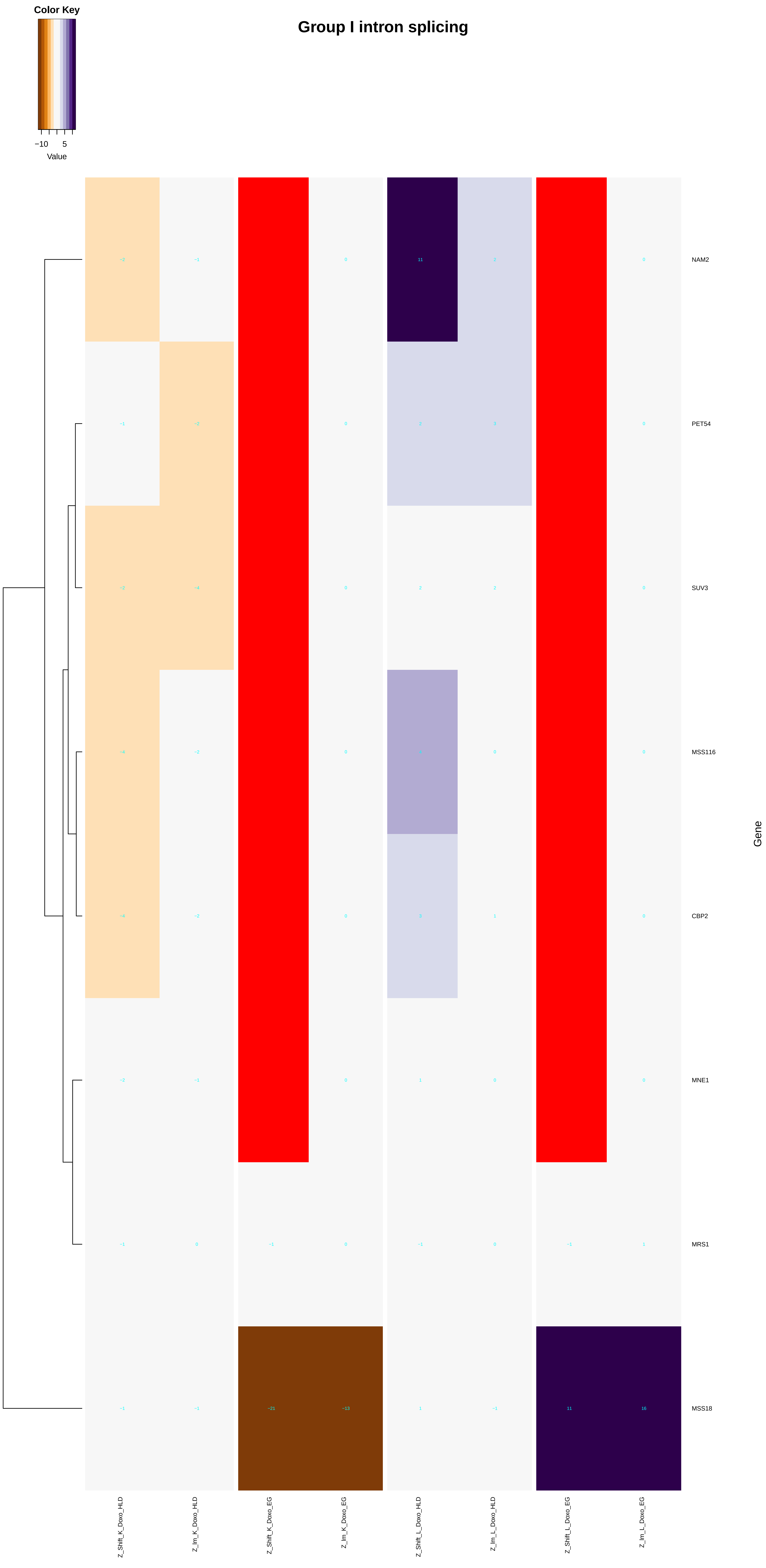

Group I intron splicing
Color Key
−10
5
Value
NAM2
PET54
SUV3
MSS116
CBP2
MNE1
MRS1
MSS18
Gene
Z_Shift_K_Doxo_HLD
Z_lm_K_Doxo_HLD
Z_Shift_K_Doxo_EG
Z_lm_K_Doxo_EG
Z_Shift_L_Doxo_HLD
Z_lm_L_Doxo_HLD
Z_Shift_L_Doxo_EG
Z_lm_L_Doxo_EG

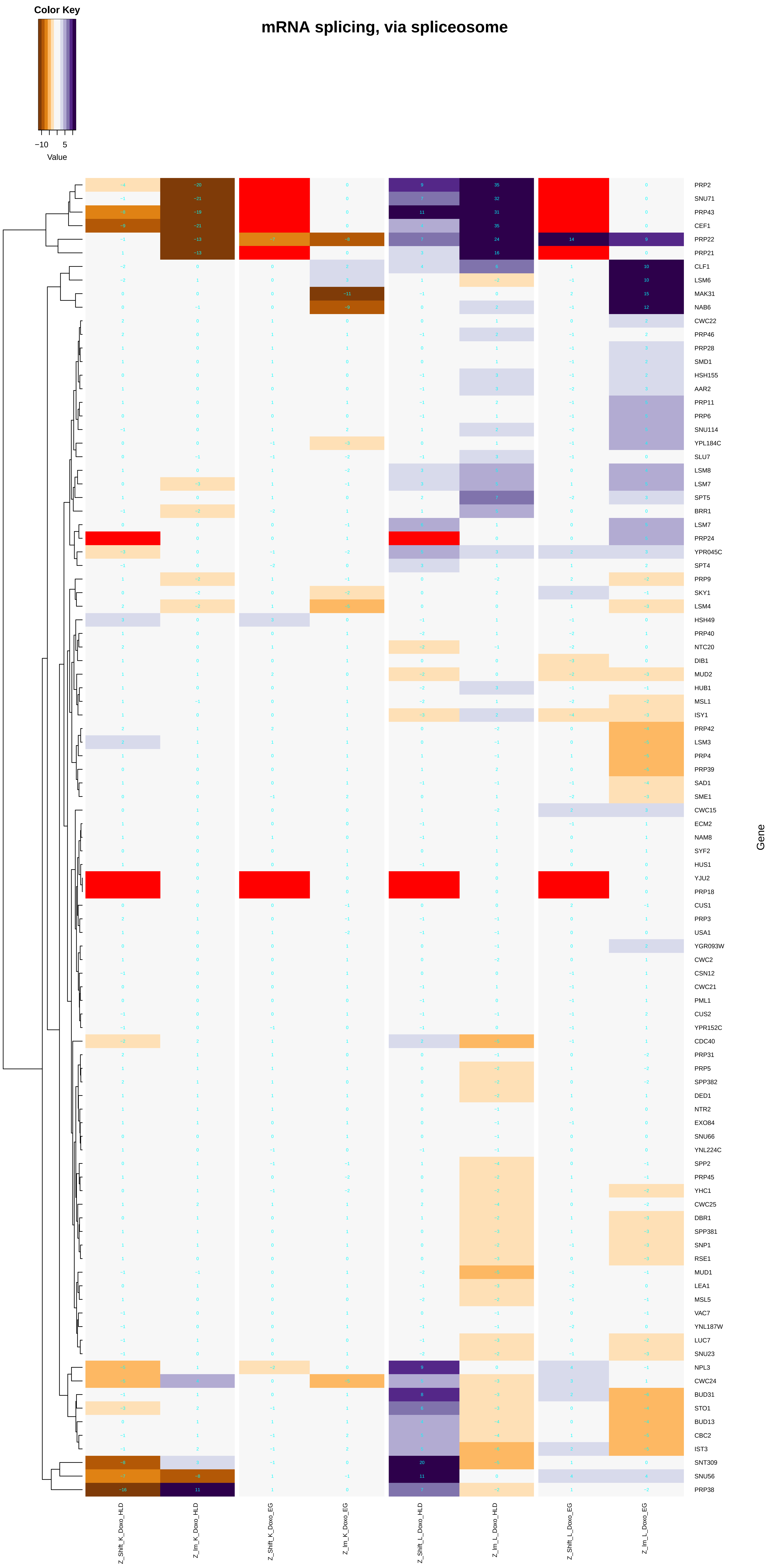
Color Key
−10
5
Value
mRNA splicing, via spliceosome
Gene
PRP2
SNU71
PRP43
CEF1
PRP22
PRP21
CLF1
LSM6
MAK31
NAB6
CWC22
PRP46
PRP28
SMD1
HSH155
AAR2
PRP11
PRP6
SNU114
YPL184C
SLU7
LSM8
LSM7
SPT5
BRR1
LSM7
PRP24
YPR045C
SPT4
PRP9
SKY1
LSM4
HSH49
PRP40
NTC20
DIB1
MUD2
HUB1
MSL1
ISY1
PRP42
LSM3
PRP4
PRP39
SAD1
SME1
CWC15
ECM2
NAM8
SYF2
HUS1
YJU2
PRP18
CUS1
PRP3
USA1
YGR093W
CWC2
CSN12
CWC21
PML1
CUS2
YPR152C
CDC40
PRP31
PRP5
SPP382
DED1
NTR2
EXO84
SNU66
YNL224C
SPP2
PRP45
YHC1
CWC25
DBR1
SPP381
SNP1
RSE1
MUD1
LEA1
MSL5
VAC7
YNL187W
LUC7
SNU23
NPL3
CWC24
BUD31
STO1
BUD13
CBC2
IST3
SNT309
SNU56
PRP38
Z_Shift_K_Doxo_HLD
Z_Im_K_Doxo_HLD
Z_Shift_K_Doxo_EG
Z_Im_K_Doxo_EG
Z_Shift_L_Doxo_HLD
Z_Im_L_Doxo_HLD
Z_Shift_L_Doxo_EG
Z_Im_L_Doxo_EG

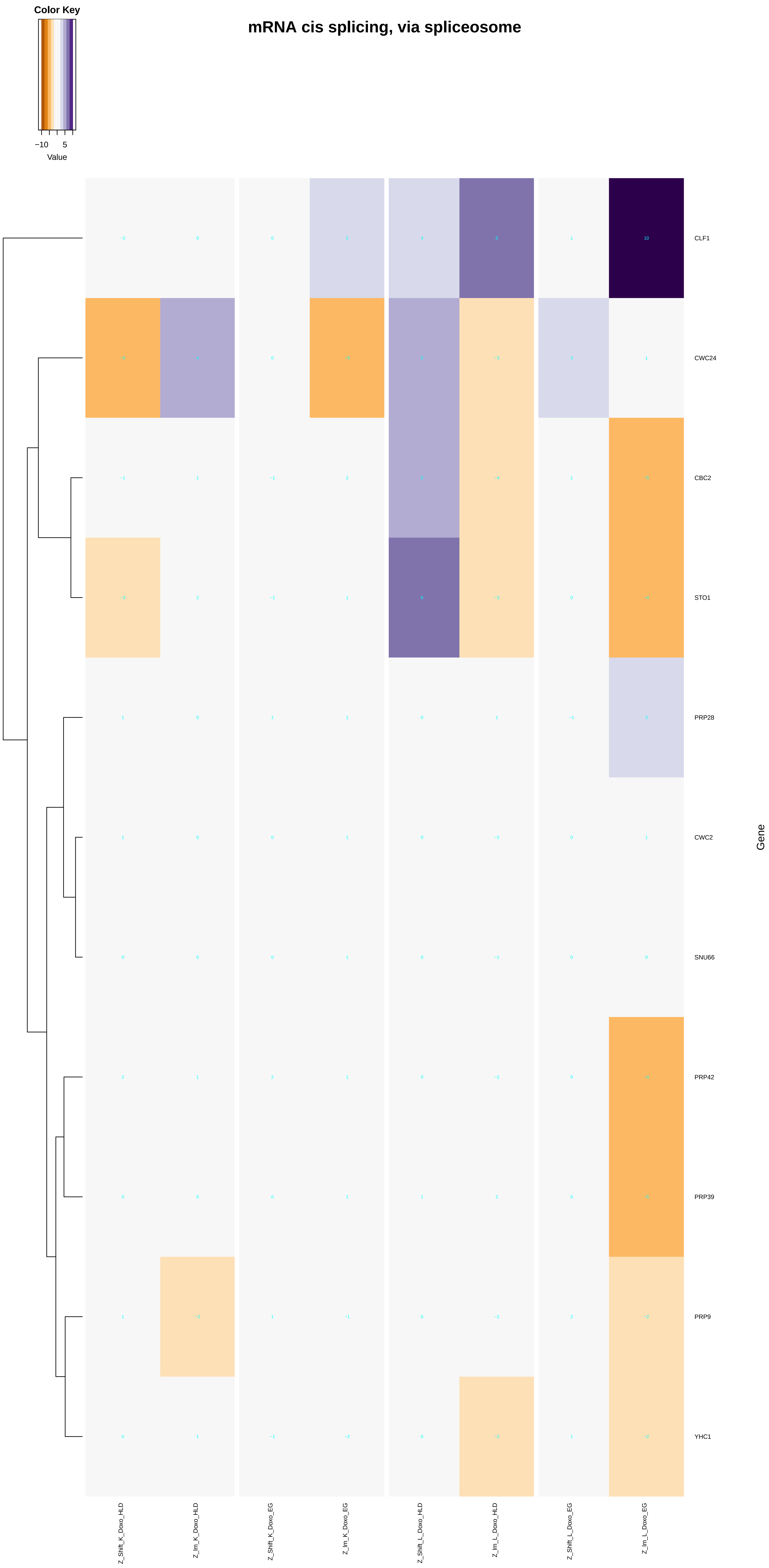
mRNA cis splicing, via spliceosome
Color Key
−10
5
Value
CLF1
CWC24
CBC2
STO1
PRP28
CWC2
SNU66
PRP42
PRP39
PRP9
YHC1
Gene
Z_Shift_K_Doxo_HLD
Z_Im_K_Doxo_HLD
Z_Shift_K_Doxo_EG
Z_Im_K_Doxo_EG
Z_Shift_L_Doxo_HLD
Z_Im_L_Doxo_HLD
Z_Shift_L_Doxo_EG
Z_Im_L_Doxo_EG
